# Library and Museum of the Medical School

**Published:** 1894-08

**Authors:** 


### Editorial Department.

F. E. DANIEL, M. D., Editor.
S. E. HUDSON, M. D., Managing Editor.
A. J. SMITH. M. D., Galveston, Associate Editor.

EDITORIAL STAFF:

PROF. J. E. THOMPSON, M. D., Texas Medical College, Galveston; Surgery.
PROF. WM. KEILLER, M. D., Texas Medical College, Galveston; Obstetrics and Gynecology.
PROF. DAVID CERNA, M. D., Texas Medical College, Galveston; Therapeutics.
PROF. A. J. SMITH, M. D., Texas Medical College, Galveston; Medicine
DR. ISADORE DYER, Tulane University; Dermatology.
DR. R. H. L. BIBB, Saltillo, Mexico; Foreign Correspondent.
DR. ROBT. MORRIS, Charity Hospital, N. O.; Clinical Reports

LIBRARY AND MUSEUM OF THE MEDICAL SCHOOL.—During the past year several hundred volumes have been added to the library of the Medical Department of the University, and the books have been arranged in the recently completed cases in the room set aside for the accommodation of the library. A card catalogue of subjects and individual articles is being made, and the working value of the collection thus greatly enhanced. It has been arranged that under proper restrictions in matter of length of loan and number of books withdrawn, any physician of Texas may take books from this library, provided he deposit the cost of same or an accredited check for the same with the librarian, and pay the cost of carriage each way. The deposit is, of course, to be returned upon the return of the books. It is hoped thus to make the library of service to the profession of the State.

In a library of as ambitious and general a scope as this, it is desirable that all sorts of books, bearing upon medical or allied science, and all editions, be deposited for consultation. Old files of medical journals, American and foreign, are especially desirable, and donations of such are eagerly desired. Any persons who are disposed to aid thus in building up a large State Medical Library, in connection with the State school, are earnestly requested to send, at the expense of the library, all volumes and files (complete or incomplete) of journals. Pack them securely, put them in charge of the express or freight company, with the address, "Library of Medical Department of University of Texas, Galveston, Texas." Their reception will be appreciated and acknowledged.

At the same time it is desirable to suggest once more the propriety of depositing in the museum all specimens of pathological interest. The present collection has grown rapidly and includes a number of rare and beautiful specimens. In such cases the name of the donor and the history of the specimen are preserved in the catalogue of the collection; and when desired, a special report will be made on the specimens sent. The school will gladly pay cost of carriage to Galveston. Address specimens to Dr. Allen J. Smith, Professor of Pathology, Medical Department University of Texas, Galveston, Texas.

